# Rectus Sheath Hematoma Due to Low Molecular Weight Heparin in a COVID-19 Patient in Turkey

**DOI:** 10.7759/cureus.14870

**Published:** 2021-05-06

**Authors:** Tolga Kalayci

**Affiliations:** 1 General Surgery, Erzurum Regional Education and Research Hospital, Erzurum, TUR

**Keywords:** anticoagulant therapy, rectus sheath hematoma, low molecular weight heparin, infection, covid-19 pneumonia, covid-19, enoxaparin sodium

## Abstract

This case report presents a large left rectus sheath hematoma (RSH) case developed in a COVID-19 patient who had received no anticoagulant therapy before hospital admission. It discusses the patient’s diagnosis and treatment process.

A 78-year-old woman was admitted to the ED with acute cough and shortness of breath. On CT scan, the pulmonary findings were consistent with COVID-19 pneumonia. Subcutaneous enoxaparin sodium was started to the patient, in accordance with the COVID-19 treatment guidelines applied in Turkey. On the ninth day after admission, her hemoglobin level decreased to 7.3 g/dL. At that point, her blood pressure was 84/52 mmHg, and her heart rate was 120 beats/min. There was a mass in the left lower quadrant^ ^on the physical exam. CT examination of the abdomen and pelvis showed a left inferior RSH approximately 9 cm wide. Enoxaparin sodium was stopped. Vital signs monitoring and fluid replacement were begun. One week after the diagnosis of RSH, a CT of the abdomen and pelvis was performed. The scan showed no significant increase in the size of the hematoma. On the 18^th^ day after admission, the patient was discharged because her hemoglobin value, which was 10.2 g/dL at that point, had not decreased, her vital signs were stable, and her treatment for COVID-19 was completed. From the moment of diagnosis to discharge, the patient required no interventional or surgical procedures.

## Introduction

Rectus sheath hematoma (RSH), a rare cause of abdominal pain, is defined as blood collected between the fibers of the rectus abdominis muscle and pyramidalis muscle [1]. RSH has many etiological factors but is commonly caused by trauma, abdominal surgery, hematological diseases, pregnancy, and physical activity. RSH can also occur after anticoagulant therapy, and early recognition of such cases can save patients’ life [2].

The COVID-19 pandemic is today’s most important problem worldwide. Although the effects on the body by COVID-19 infection are unknown, studies have shown that it can trigger venous thromboembolic events [3]. Vascular injury in the kidneys, liver, and spleen has also been seen in COVID-19 patients [4]. The exact mechanism causing this vascular injury is not yet truly understood, but it has been hypothesized that the process is a distinct one unique to the severe acute respiratory syndrome coronavirus 2 (SARS-CoV-2) virus [5]. Therefore, according to the "Anticytokine-Anti-inflammatory Therapies, Coagulopathy Management" guideline published by the Ministry of Health, Republic of Turkey on 02.11.2020, it is recommended to use enoxaparin 40 mg 2 x 1 subcutaneously in patients with a body mass index (BMI) of more than 40 for prophylaxis [6].

RSH after anticoagulant use has been frequently reported worldwide [7-9]. To date, there have been two prior reports of RSH secondary to COVID-19 coagulopathy [6, 10]. However, no studies have been published on this subject in Turkey.

This case report presents a large left RSH case in a COVID-19 patient and discusses the patient’s treatment process.

## Case presentation

A 78-year-old woman, who had a history of hypertension and diabetes mellitus and no history of surgery, was admitted to the ED of Erzurum Regional Education and Research Hospital, Erzurum, Turkey, in December 2020 with acute cough and shortness of breath for three days. The patient had no history of anticoagulant therapy use. On evaluation, the patient’s vital findings were as follows: blood pressure, 103/52 mmHg; pulse rate, 108 beats/ min (bpm); oxygen saturation on room air, 88%‒90%; respiratory rate, 24 times/min; and fever, 37.0°C. Auscultation of the lungs revealed only wheezing. An abdominal physical examination was benign. The patient's weight was 107 kg, and her height was 160 mm (BMI of the patient = 41.79).

**Figure 1:**
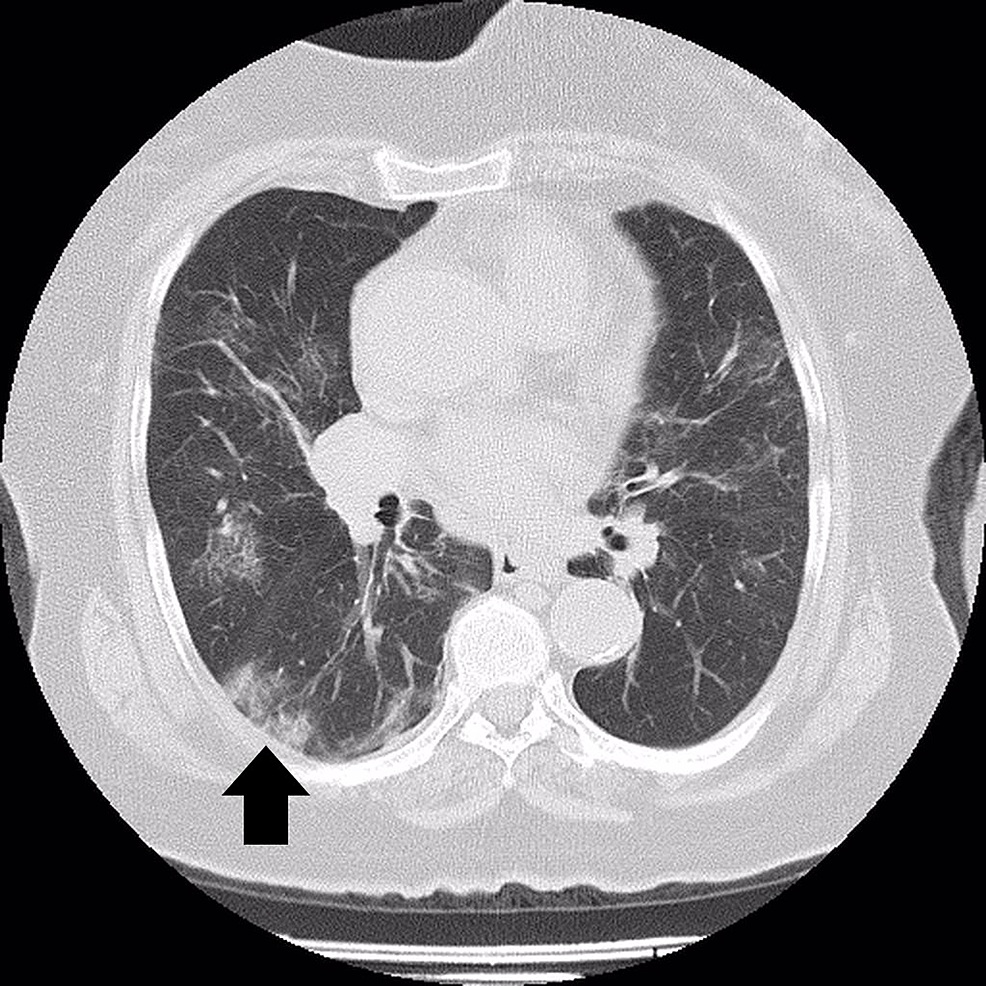
A 78-year-old woman patient with COVID-19 pneumonia (areas with pneumonitis are shown with an arrow).

The patient’s levels of C-reactive protein (CRP) (63 mg/L), lactate dehydrogenase (320 U/L), and D-dimer (995 ng/mL) were elevated. The results of other laboratory tests were unremarkable. The patient’s hemoglobin level was 13.4 g/dL. On CT scan, the pulmonary findings were consistent with COVID-19 pneumonia (Figure 1). Therefore, the patient was admitted for follow-up, and treatment was begun using favipiravir 200 mg tablets (four tablets per day), moxifloxacin 400 mg tablets (one tablet per day), and piperacillin-tazobactam 4.5 g IV (four vials per day). In addition, enoxaparin sodium (40 mg / 0.4 mL subcutaneously every 12 h) was started as prophylactic dose based on BMI of the patient.

**Figure 2:**
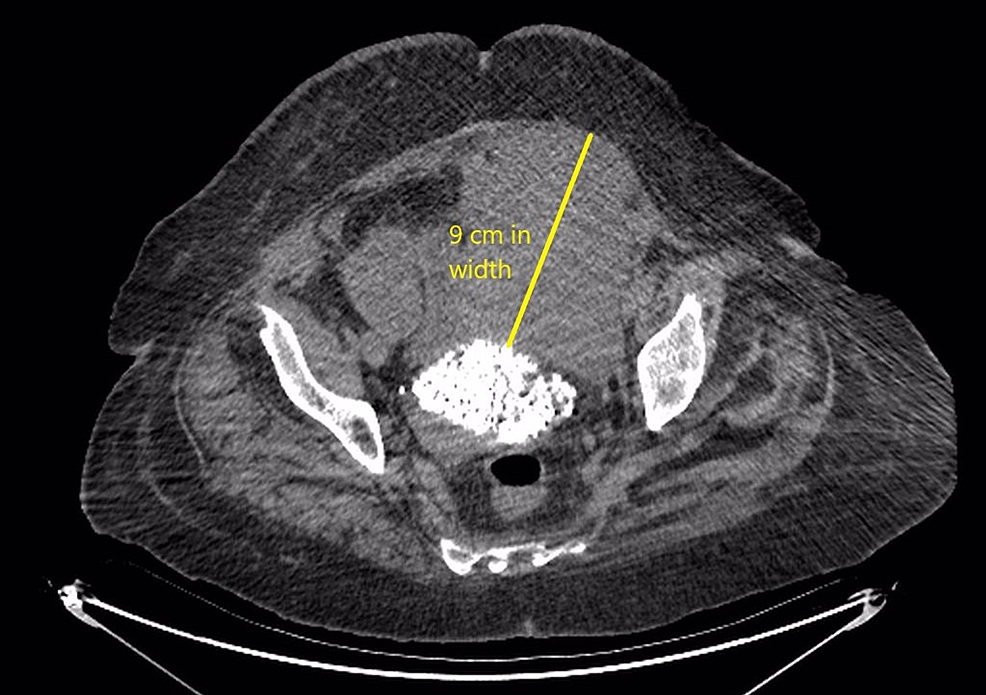
Abdominal and pelvic CT showed a left inferior rectus sheath hematoma reaching approximately 9 cm in width.

On the ninth day of the patient’s follow-up, her hemoglobin levels decreased to 7.3 g/dL. At that point, her blood pressure was 84/52 mmHg, and her heart rate was 120 bpm. An abdominal exam revealed a mass in the left lower quadrant. CT scan of the abdomen and pelvis showed a left inferior RSH approximately 9 cm wide (Figure 2).

Anticoagulation therapy was stopped. Vital signs monitoring and fluid replacement were begun. The goal was to maintain hemoglobin value above 7 g/dL. As the hemoglobin value of the patient fell below 7 g/dL twice, four units of erythrocyte suspension (ES) was administered to the patient. Abdominal examination was performed daily. One week after the diagnosis of RSH, a CT of the abdomen and pelvis was performed. The scan showed no significant increase in the size of the hematoma. On the 18th day after admission, the patient was discharged because her hemoglobin value, which was 10.2 g/dL at that point, had not decreased, her vital signs were stable, and her treatment for COVID-19 was completed. From the moment of diagnosis to discharge, the patient required no interventional or surgical procedures.

## Discussion

COVID-19 infection is well known to cause increased coagulopathy, with pulmonary emboli being the most common presentation [11-12]. Increased levels of D-dimer and fibrinogen degradation products are associated with poor prognosis and are potentially related to the risk of disseminated intravascular coagulation [12]. Therefore, according to the "Anti cytokine-Anti-inflammatory Therapies, Coagulopathy Management" guideline published by the Ministry of Health, Republic of Turkey on 02.11.2020, it is recommended to use enoxaparin 40 mg subcutaneously every 12 h in nonsevere COVID-19 patients with a BMI of more than 40 for prophylaxis [6]. While prophylactic doses of anticoagulant therapy are recommended for patients who are followed in the service, the use of anticoagulant drugs at therapeutic dose is recommended for patients who are followed up in the ICU [13-15]. A study from China suggests that in the absence of venous thromboembolism (VTE) prophylaxis, 25% of COVID-19 patients developed deep vein thrombosis (DVT), which is higher than the 5%-15% incidence seen in placebo arms of early studies of VTE prevention in medically ill hospitalized patients [16]. In the study of Moores et al., it was identified that thrombotic events in 7.7% of patients admitted with COVID-19, estimated a cumulative rate of 21% [17]. Also, Barnes et al. recommended anticoagulant prophylaxis for patients with COVID-19 when hospitalized [13].

An uncommon complication of anticoagulation therapy is RSH, a potentially life-threatening complication [1]. Early diagnosis and intervention are key to decreasing patient mortality and morbidity [18].

This case report presents a large left RSH case in a COVID-19 patient and discusses the patient’s treatment process. For diagnosis, careful physical examination is important and can detect ecchymotic areas of the abdominal skin and palpable painful masses. To confirm the diagnosis, we recommend contrast-enhanced CT, unless it is contraindicated. A CT scan with contrast also provides information about contrast extravasation and all the intra-abdominal structures. However, in cases in which contrast is contraindicated, noncontrast CT is also helpful.

After diagnosing RSH in a patient due to the administration of enoxaparin sodium, we recommend the immediate discontinuation of enoxaparin sodium. The patient’s vital signs should also be monitored closely. Each day, the abdomen should be examined, and hemoglobin levels should be checked frequently (every six hours in the first days after diagnosis). If patients have hemoglobin values lower than 7 g/dL, the goal should be to increase the value above this level if possible and maintain it. The need for ES should be evaluated according to the hemoglobin value.

In patients who have had no sudden decrease in hemoglobin values during follow-up and whose vital signs are stable, we recommend the use of noncontrast abdominal and pelvic CT one week after RSH diagnosis to evaluate whether there has been an increase in the hematoma’s size. If there is no increase in size and the patient’s hemoglobin value has not decreased by the end of the week of follow-up, patients whose vital signs are stable can be discharged and called back for a check on the third day after discharge. However, patients who have experienced even a moderate increase in the size of the hematoma should continue until hemodynamic stability is achieved.

## Conclusions

In conclusion, a healthcare provider administering subcutaneous anticoagulant therapy such as enoxaparin sodium should be aware of potential complications, and RSH should be suspected in the differential diagnosis of any patient complaining of abdominal pain after a subcutaneous injection. Further research and case reports on acute hemorrhage in COVID-19 patients receiving anticoagulant therapy for prophylaxis are recommended.
